# Characteristics and Obstetric Outcomes in Women With Autoimmune Rheumatic Disease During the COVID-19 Pandemic in Qatar

**DOI:** 10.7759/cureus.24382

**Published:** 2022-04-22

**Authors:** Eman Satti, Monika Ostensen, soha darrgham, Nawal Hadwan, Hadeel Ashour, Samar AL Emadi

**Affiliations:** 1 Intenal Medicine, Rheumatology Section, Hamad Medical Corporation, Doha, QAT; 2 Department of Rheumatology, Sorlandet Hospital, Kristiansand, NOR; 3 Biostatistics, Epidemiology, and Biomathematics Research Core, Weill Cornell Medicine, Doha, QAT; 4 Internal Medicine, Rheumatology Section, Hamad Medical Corporation, Doha, QAT

**Keywords:** autoimmune rheumatic diseases, caesarean, covid-19, prematurity, pregnancy

## Abstract

Objective: Pregnant women with autoimmune rheumatic diseases are considered to have a high risk of obstetric complications with the emergence of the Coronavirus disease (COVID-19) pandemic. Therefore, we aimed to assess the impact of COVID-19 on this high-risk group.

Methods: This cross-sectional cohort study (March to December 2020) was conducted at the largest tertiary center in Qatar (Hamad Medical Corporation). Eighty consecutive patients following up at the center during pregnancy were surveyed through telephonic interviews. Data on COVID-19 and pregnancy outcomes were extracted from electronic hospital records.

Results:Eighty pregnant women with autoimmune rheumatic diseases were included. Among them, 17 (21.3%) (95% confidence interval [CI]: 12.9-31.8%) were diagnosed with COVID-19, five were hospitalized, and only one required intensive care unit admission. The proportion of adverse obstetric outcomes in the cohort was 29.5% (n = 23; 95% CI: 19.7-40.9%). Prematurity (n = 14; 19.4%) and caesarean section (n = 30; 41.1%) were the most prevalent adverse events. There was no statistical difference in adverse pregnancy outcomes between women with and without COVID-19.

Conclusion: COVID-19 did not affect pregnancy outcomes in women with autoimmune rheumatic diseases.

## Introduction

Since the emergence of severe acute respiratory syndrome coronavirus 2 (SARS-CoV-2) causing the coronavirus disease (COVID-19) pandemic, pregnant women have received particular attention for being at high risk for increased morbidity and mortality. A systematic review analysed 198 studies (December 2019 to June 2020) and found that pregnant women are less likely to develop clinical symptoms of COVID-19 compared to non-pregnant women but have higher odds for intensive care admissions (odds ratio [OR] 2.13, 95% confidence interval [CI] = 1.53-2.95; I2 = 71.2%) and invasive ventilation (OR = 2.59, 95% CI = 2.28-2.94; I2 = 0%). The most prominent perinatal adverse event in women with COVID-19 is preterm delivery (OR = 1.47, 95% CI = 1.14-1.91; I2 = 18.6%) [1].

Pregnancy in women with autoimmune rheumatic diseases such as systemic lupus erythematosus (SLE), Sjögren syndrome, and inflammatory arthropathies is associated with a risk of adverse outcomes, including miscarriage, intrauterine foetal death, pre-eclampsia, prematurity, and neonatal morbidities [2]. In addition, patients with autoimmune rheumatic diseases are at high risk of developing severe COVID-19 infection owing to altered immune responses and treatment with immunosuppressants. A matched cohort study of COVID-19 infection found that the rate of intensive care admission and mechanical ventilation in patients with rheumatic diseases was three times that of patients with non-rheumatic diseases (OR 3.11, 95% CI = 1.07-9.05) [3]. However, exposure to different classes of disease-modifying antirheumatic drugs (DMARDs) did not increase the risk of hospitalisation, except for corticosteroids (≥10 mg/day) [4].

A web-based survey study from a single centre in New York evaluated the impact of COVID-19 on pregnant patients with rheumatic diseases and reported a similar prevalence among pregnant and non-pregnant women reporting to the centre. It also found that prenatal care was impacted in the majority of these patients [5].

Bermas et al. examined data from the COVID-19 Global Rheumatology Alliance registry and assessed pregnancy outcomes in 22 patients with rheumatic diseases who were pregnant when they encountered the infection. Only 10 pregnant women needed hospitalisation, and all recovered from the infection. They also reported favourable pregnancy outcomes with 16/22 term births, one miscarriage, and one termination at nine weeks [6].

At present, there is a paucity of data regarding the impact of COVID-19 on pregnant women with autoimmune rheumatic diseases [7]. Thus, we examined the maternal and foetal outcomes in this high-risk group of patients during the first wave of the COVID-19 pandemic in Qatar.

## Materials and methods

This cross-sectional study was conducted at the largest tertiary health care centre in Qatar (Hamad Medical Corporation) in compliance with the Helsinki Declaration and was approved by the Medical Research Centre and Institutional Review Board at Hamad Medical Corporation (MRC-01-20-617).

We screened 80 pregnant women who visited a specialised "Pregnancy and Rheumatic Disease Clinic" at least once, from the start of the COVID-19 pandemic in Qatar until the end of the recruitment period (March 20 to December 20, 2020). Pregnant patients diagnosed with autoimmune rheumatic disease according to international criteria (ACR/EULAR) by a rheumatologist and with a singleton pregnancy were followed up once in each trimester and once postpartum. Follow-up data included baseline demographics (age, smoking, baseline body mass index), diagnosis, medical history, comorbidities, medication during pregnancy, obstetric and perinatal outcomes, gestational age (weeks), birth weight (kilograms), neonatal intensive care unit (NICU) admission, mode of delivery, and congenital malformations. Obstetric morbidities including pre-eclampsia, gestational diabetes, premature rupture of the membranes (PROM), stillbirth, and miscarriage were diagnosed based on the ACOG (American College of Obstetricians and Gynecologists) definitions. Prematurity was defined as delivery before 37 weeks of gestation, and low birth weight was defined as birth weight less than 2.5 kg. The treating physicians documented the standard data on disease activity, pregnancy, and peripartum outcomes from the hospital's electronic records.

Disease activity was measured using the following scores: (a) for rheumatoid arthritis, the Clinical Disease Activity Index score of ≤3 for remission and >3 for active disease; (b) for lupus, the SLE Disease Activity Index score was adjusted for pregnancy, and a score of >3 was used to stratify "active disease"; (c) for the other connective tissue/inflammatory diseases (Sjögren syndrome, scleroderma, Behcet’s disease, and Familial Mediterranean Fever), patient and physician global assessment were used. Remission was defined as patient global assessment ≤1 using a 0-10 visual analogue scale. Independent from transient flares, a disease was recorded as "active" if it required therapy escalation and remission was not achieved by the end of pregnancy.

Eighty consecutive pregnant patients were approached and informed about this COVID-19 study. Eligibility criteria include any woman previously diagnosed with an autoimmune rheumatic disease and who is currently pregnant with a singleton. All patients were eligible and agreed to participate. After obtaining verbal consent, the participants completed a telephonic survey questionnaire that included baseline characteristics, medical history, comorbidities, medications used during pregnancy, history of travel, and contact with confirmed COVID-19 cases. They were provided with a hotline number to report whether they had been diagnosed or had been in close contact with any household or co-worker at the same office with COVID-19 positivity or infection. The COVID-19 diagnosis was only confirmed based on a positive nasopharyngeal polymerase chain reaction (PCR). The survey data has been updated with the most recent information.

At the completion of the recruitment period, two investigators (NH and HA) systematically extracted prespecified clinical variables, including disease activity measures in the third trimester, medications during pregnancy, pregnancy morbidities, peripartum outcomes, and the course of COVID-19 infection, from the electronic records of all participants. Data from the surveys and the electronic records were then linked and analysed.

**Figure 1:**
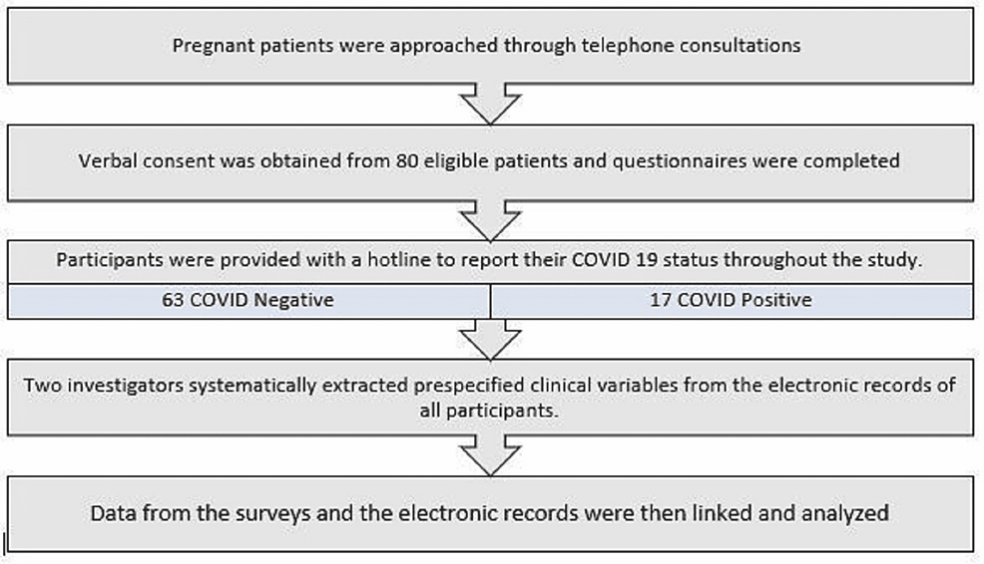
Study methodology flowchart.

Statistical methods

Sample characteristics are presented as frequency distributions for categorical variables using the chi-square test and means and standard deviations for continuous variables using the t-test. Statistical significance was defined as p < 0.05. All analyses were performed using IBM SPSS Statistics for Windows, version 26.0 (IBM Corp., Armonk, NY, USA).

## Results

Eighty pregnant women with rheumatic diseases, who completed their pregnancy during the study period, were included in this analysis. Table 1 presents the baseline characteristics of the participants. The distribution of primary diagnoses, disease duration (p = 0.99), and disease activity (p = 0.63) were not significantly different between pregnant women who had COVID-19 infection and those without COVID-19 (p > 0.09). Although both groups were similar in their medication profiles, hydroxychloroquine intake was significantly higher in women without COVID-19 (p = 0.04).

**Table 1:** Baseline characteristics of pregnant women during the COVID-19 pandemic. COVID-19: Coronavirus disease, IQR: interquartile range, SLE: systemic lupus erythematosus, TNF: tumour necrosis factor Spondylarthritis includes ankylosing spondylitis, psoriatic arthritis, peripheral spondylarthritis; other: Behcet’s syndrome, familial Mediterranean fever

	Mother's COVID-19 status		P-value
	Negative (63)	Positive (17)	
	n (%)	n (%)	
Age (years); median (IQR)	34.0 (28.0–36.0)	31.0 (29.0–33.5)	0.231
Morbidities			
Hypertension	3 (4.8)	1 (5.9)	0.851
Diabetes	2 (3.2)	1 (5.9)	0.517
Hypothyroidism	9 (14.3)	2 (11.8)	0.786
Disease profile			
Primary diagnosis			
SLE	20 (31.7)	4 (23.5)	>0.099
Sjögren syndrome	16 (25.4)	3 (17.6)	
Systemic sclerosis	0 (0.0)	1 (5.9)	
Antiphospholipid syndrome	6 (9.5)	2 (11.8)	
Ankylosing spondylitis	3 (4.8)	1 (5.9)	
Psoriatic arthritis	2 (3.2)	0 (0.0)	
Behcet's syndrome	1 (1.6)	0 (0.0)	
Peripheral SPA	0 (0.0)	2 (11.8)	
Inflammatory diseases	0 (0.0)	1 (5.9)	
Disease duration (years); median (IQR)	4.0 (2.0–8.0)	5.0 (1.3–6.0)	0.991
Disease activity			
Remission	58 (92.1)	15 (88.2)	0.636
Active	5 (7.9)	2 (11.8)	
Medication			
Hydroxychloroquine	53 (84.1)	10 (58.8)	0.041
Sulfasalazine	11 (17.5)	3 (17.6)	0.986
Azathioprine	8 (12.7)	5 (29.4)	0.136
Glucocorticoids	8 (12.7)	3 (17.6)	0.693
Anti-TNF	8 (12.7)	2 (11.8)	0.918
Close contact with someone diagnosed with COVID-19	9 (14.3)	8 (47.1)	0.007

Pregnancy and neonatal outcomes

Results of the univariate analysis for pregnancy and neonatal outcomes are presented in Table 2. The rates of pregnancy complications and adverse neonatal outcomes were low. Considering that some women experienced overlapping pregnancy and neonatal complications, the proportion with a complicated outcome was 29.5% (n = 23; 95% CI = 19.7-40.9%) versus 70.5% (n = 55; 95% CI = 59.1-80.3%) with uncomplicated outcomes. Prematurity (n = 14; 19.4%) and caesarean section (n = 30; 41.1%) were the most prevalent adverse events. Demographic characteristics, disease profile, and use of rheumatic medications were not significantly associated with pregnancy and neonatal outcomes.

**Table 2:** Obstetric and foetal complications in women with rheumatic diseases. *Complex cardiac anomaly; COVID-19: coronavirus disease, PROM: premature rupture of membranes, prematurity (27–37 weeks), NICU: neonatal intensive care unit, NVD: normal vaginal delivery, CS: caesarean section, assisted delivery: delivery by vacuum or forceps.

	Mother's COVID-19 status		P-value
	Negative (63)	Positive (17)	
	n (%)	n (%)	
Gestational diabetes	3 (4.8)	2 (11.8)	0.286
Eclampsia	1 (1.6)	1 (10.0)	0.257
Foetal complication			
Miscarriage	4 (6.3)	1 (6.7)	0.783
Stillbirth	2 (3.2)	0 (0.0)	
Healthy	57 (90.5)	14 (93.3)	
Pregnancy term			
Term	45 (77.6)	14 (93.3)	0.276
Preterm	13 (22.4)	1 (7.1)	
PROM			
Yes	5 (7.9)	0 (0.0)	0.394
N/A	8 (12.7)	1 (6.7)	
Low birthweight	9 (16.1)	1 (7.1)	0.674
Congenital abnormalities*	1 (1.8)	0 (0.0)	>0.999
NICU admission	5 (8.8)	1 (7.1)	>0.999
Mode of delivery			
NVD	30 (50.8)	6 (42.9)	0.757
C/S	24 (40.7)	6 (42.9)	
Assisted delivery	5 (8.5)	2 (14.3)	

COVID-19 during pregnancy

The prevalence of COVID-19 among this study's sample was 21.3% (95% CI = 12.9-31.8%). At the time of the SARS-CoV-2 PCR, most patients were asymptomatic (90%); however, all patients developed mild symptoms later during the illness.

Fever was the most reported symptom (11 patients), followed by cough and diarrhoea (eight patients and one patient, respectively). Only two patients experienced dyspnoea, and two had anosmia. Five patients were hospitalised, and only one required oxygen therapy and intensive care admission. All patients continued using their antirheumatic medication during the infection period until full recovery.

## Discussion

This study is one of the first few studies to investigate the pregnancy outcomes in pregnant women with rheumatic diseases who had COVID-19 infection and those without COVID-19 infection. We identified a higher prevalence of COVID-19 among our sample (21.3%) than the previously reported 10% in pregnant and peripartum women [1,8]. The infection occurred mostly in the second and third trimesters of pregnancy. Most women were tested because of contact tracing after close contact with an infected patient. The majority were asymptomatic at the time of PCR; however, all of them developed mild symptoms later. This supports the findings of a systematic review of 192 studies showing that pregnant women are less likely to have symptoms of COVID-19 than non-pregnant women [1]. Another study showed that pregnant women with rheumatic diseases had more frequent COVID-19 symptoms but with shorter disease duration [5].

Patients with rheumatic diseases, in general, have an increased risk of intensive care unit (ICU) admission and mechanical ventilation [3]. Among pregnant women in the general population, comorbidities and pre-eclampsia increase the risk of ICU admission, mechanical ventilation, and severe COVID-19 infection [1]. Fever and cough were the predominant symptoms in our pregnant patients, and only a minority needed hospitalization. A similar finding was observed in data from the Global COVID-19 Registry, where only 5% of pregnant women with autoimmune rheumatic diseases required hospitalization with supplemental oxygen, and all eventually recovered [6].

The Society for Obstetric Anaesthesia and Perinatology (SOAP) COVID-19 Registry studied the peripartum outcomes in women who had COVID-19 infections and those without COVID-19 and found that most patients with the infection were asymptomatic (61.1%). Patients with symptomatic infection carried an increased risk for caesarean section, increased postpartum length of stay, and premature delivery [9]. In our study, we did not find a link between the development of symptoms and adverse pregnancy outcomes. This information may be subject to recall bias and needs to be explored in further studies. The rates of caesarean section and prematurity were similar to the data reported by the COVID-19 Global Registry Alliance (GRA) [6].

In our cohort, six of the very premature children required NICU admission but recovered completely. Neonates born to mothers who were and were not previously diagnosed with COVID-19 were not routinely tested using PCR. Thus, the COVID-19 status of the newborns remained unknown. One infant was diagnosed with COVID-19 at two months of age after being in close contact with his grandmother and mother, both of whom were diagnosed with the disease. He remained asymptomatic while his mother continued breastfeeding and using her antirheumatic medications. Nevertheless, a previous study has shown that vertical transmission of COVID-19 is extremely low [10].

Although some DMARDs have been promising in the treatment of COVID-19 infection cases and could have a role in the prevention of cytokine storms [11], it is still recommended to withhold them for confirmed COVID-19 infection cases [12]. None of the pregnant patients in our cohort were recommended to modify or withhold their treatment during the infection period because they did not self-report to their rheumatologist at the time of infection; they continued their antirheumatic medications throughout pregnancy and during their COVID-19 infection without developing infection-related complications, supporting the observation in the GRA that DMARDs did not increase the risk for hospitalization, except for glucocorticoids [4]. Although disease activity measures were recorded using standardized methods [13-15], the impact of disease activity on the outcome of pregnancy or COVID-19 infection could not be assessed in this study.

The limitations of this study include the dependence on the patients’ ability to recall symptoms of infection and a history of close contact, which makes the findings related to these two parameters relatively inaccurate in spite of our attempt to provide patients with hotline access to report this information. The remaining data related to pregnancy outcomes were confirmed by hospital electronic records and were not sensitive to recall bias. Further limitations include the small sample size, lack of a control group of healthy pregnant women, and the discrepancy in sample size between the two groups, rendering it challenging to deduce the impact of the underlying autoimmune rheumatic disease, independent of the impact of COVID-19, on the pregnancy outcome.

## Conclusions

This is the first study in Qatar to report the outcome of pregnancy in patients with autoimmune rheumatic diseases during the COVID-19 pandemic. Despite the high proportion of COVID-19 among these patients, neither the presence of symptoms nor the underlying rheumatic disease or its treatment resulted in increased adverse maternal or pregnancy outcomes. This observation may reassure pregnant patients with rheumatic diseases during the pandemic. Nevertheless, larger prospective cohorts and matched non-rheumatic disease (healthy pregnant) controls are required to further assess the impact of antirheumatic medications and disease activity on pregnancy outcomes in patients with COVID-19.
